# Breaking down barriers to COPD management in primary care: applying the updated 2023 Canadian Thoracic Society guideline for pharmacotherapy

**DOI:** 10.3389/fmed.2024.1416163

**Published:** 2024-08-06

**Authors:** Alan Kaplan, Amanda Babineau, Robert Hauptman, Suzanne Levitz, Peter Lin, Molly Yang

**Affiliations:** ^1^Family Physician Airways Group of Canada, University of Toronto, Toronto, ON, Canada; ^2^Respiratory Health Clinic, Vitalité Health Network, Moncton, NB, Canada; ^3^Family Physician Airways Group of Canada, Department of Family Medicine, University of Alberta, Edmonton, AB, Canada; ^4^Medical Director Inpatient Pulmonary Rehabilitation Program, Mount Sinai Hospital, Montreal, QC, Canada; ^5^Director Primary Care Initiatives, Canadian Heart Research Centre, Toronto, ON, Canada; ^6^Wholehealth Pharmacy Partners, Markham, ON, Canada

**Keywords:** COPD, primary care, guidelines, implementation, barriers

## Abstract

Chronic obstructive pulmonary disease (COPD) is a highly prevalent yet under-recognized and sub-optimally managed disease that is associated with substantial morbidity and mortality. Primary care providers (PCPs) are at the frontlines of COPD management, and they play a critical role across the full spectrum of the COPD patient journey from initial recognition and diagnosis to treatment optimization and referral to specialty care. The Canadian Thoracic Society (CTS) recently updated their guideline on pharmacotherapy in patients with stable COPD, and there are several key changes that have a direct impact on COPD management in the primary care setting. Notably, it is the first guideline to formally make recommendations on mortality reduction in COPD, which elevates this disease to the same league as other chronic diseases that are commonly managed in primary care and where optimized pharmacotherapy can reduce all-cause mortality. It also recommends earlier and more aggressive initial maintenance inhaler therapy across all severities of COPD, and preferentially favors the use of single inhaler therapies over multiple inhaler regimens. This review summarizes some of the key guideline changes and offers practical tips on how to implement the new recommendations in primary care. It also addresses other barriers to optimal COPD management in the primary care setting that are not addressed by the guideline update and suggests strategies on how they could be overcome.

## 1 Introduction

Chronic obstructive pulmonary disease (COPD) is a prevalent chronic respiratory disease that remains a leading cause of morbidity and mortality (1). Concerningly, hospitalizations for COPD have continued to increase in Canada over the last 15 years even after controlling for population growth and the aging population and despite declining rates of cigarette smoking (2). In contrast, hospital admissions for other chronic diseases have declined in the same period (2), underscoring the high burden of COPD on the healthcare system and the opportunity to improve patient outcomes.

Primary care providers (PCPs), including family physicians (FPs), nurses, respiratory therapists (RTs), certified respiratory educators (CREs), and pharmacists, are at the frontlines of the management of COPD. Indeed, a Canadian cross-sectional study suggests that the majority of ambulatory COPD patients are managed in the primary care setting and that only a minority (11%) consulted a respirologist (3). Even among individuals hospitalized for an acute exacerbation of COPD (AECOPD), less than one-third (30%) saw a respirologist (3). Therefore, PCPs play an integral role across all stages of COPD management including diagnosis, monitoring disease progression, identification and treatment of exacerbations, assessment of medication adherence, device instruction, disease education and self-management, individualized action plans, and appropriate referrals to specialist care (4).

Recently, the Canadian Thoracic Society (CTS) updated their guideline on pharmacotherapy in patients with stable COPD (5), which updates their previous 2019 guideline (6). The CTS guideline is an important tool that supports PCPs in delivering optimal pharmacological therapy for individuals with COPD that is founded on the latest scientific evidence. Importantly, this internationally recognized guideline can help PCPs address gaps in the clinical care of patients with COPD by offering evidence-based treatments aimed at relieving dyspnea, improving overall health status, preventing exacerbations, and, for the first time, reducing mortality in patients with COPD (5).

This review aims to update PCPs on the optimal management of COPD through the lens of key changes in the 2023 CTS guideline and to propose pragmatic solutions to address challenges to their implementation in routine practice. It also aims to propose solutions to other barriers that cannot be adequately addressed by pharmacotherapeutic guidelines but that nonetheless have an important impact on patient care and outcomes.

## 2 Assessment of key changes to the 2023 CTS guideline on pharmacotherapy for stable COPD and implications

Since the publication of the 2019 CTS guideline on COPD pharmacotherapy, the body of literature on the pharmacological management of COPD has expanded considerably. Indeed, through a comprehensive and systematic search of the published literature, the 2023 CTS guideline committee identified more than one-thousand records, of which 203 original citations were included (5). While PCPs are encouraged to consult the full 2023 CTS guideline for a detailed summary of the recommendations and supporting evidence, this review will focus on three key changes that have direct actionable impact on the optimization of primary care management of COPD.

### 2.1 Key change #1: simplified algorithm for COPD pharmacotherapy

**Figure 1:**
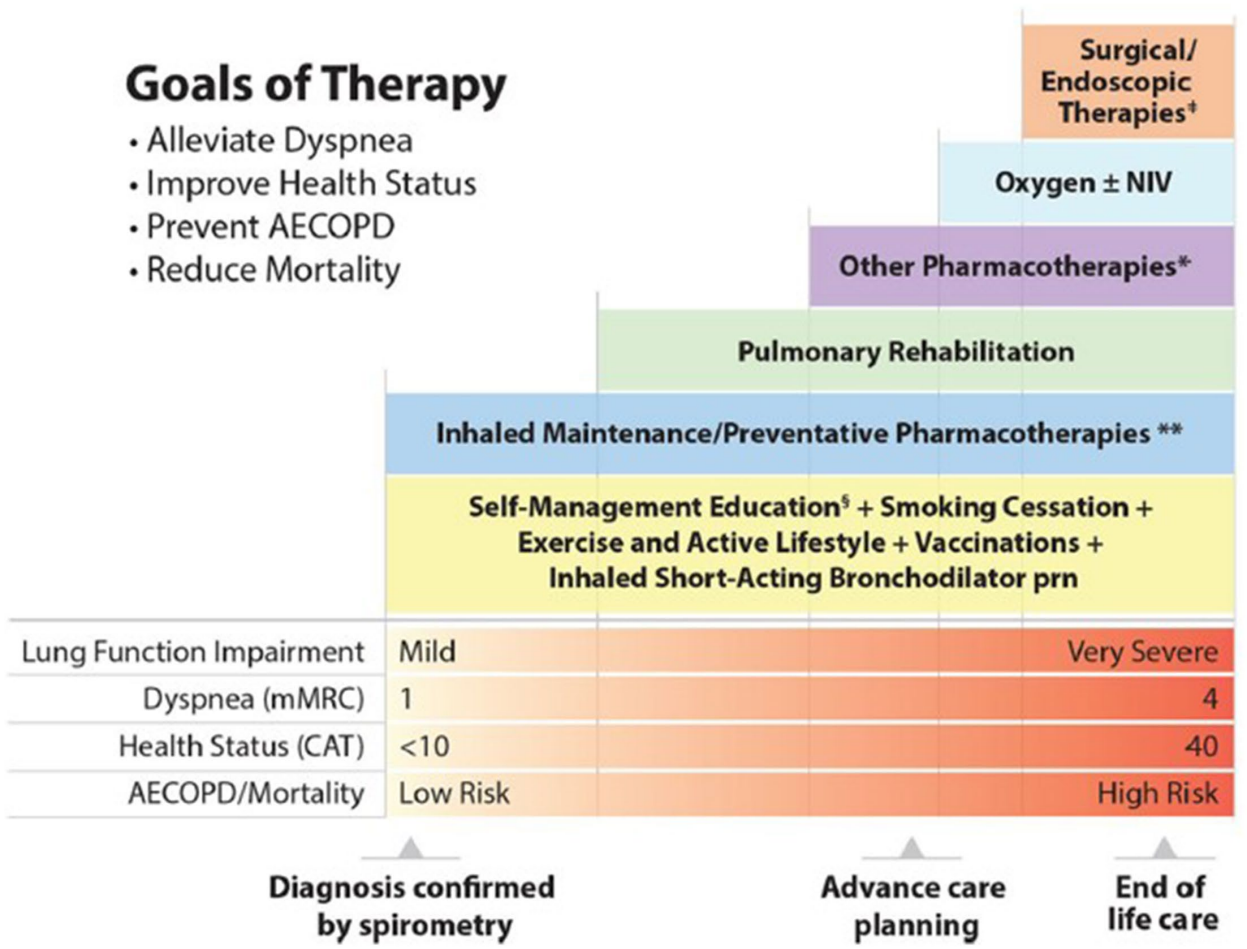
2023 CTS guideline: Integrated comprehensive management of COPD (5). Reprinted by permission of Taylor & Francis Ltd. (https://www.tandfonline.com) on behalf of 2023 Canadian Thoracic Society, [from Bourbeau et al. (5)]. AECOPD, acute exacerbation of COPD; CAT, COPD assessment test; mMRC, Modified Medical Research Council; NIV, noninvasive ventilation. *Other pharmacotherapies include oral therapies (prophylactic macrolide, and PDE-4 inhibitor, mucolytic agents for patients with chronic bronchitis), alpha-1-antitrypsin augmentation therapy for documented severe A1AT deficiency, and opioids for severe refractory dyspnea. ^**^Inhaled Maintenance/Preventative Pharmacotherapies are long-acting muscarinic antagonists (LAMA) and/or long-acting ẞ2-agonists (LABA) with or without inhaled corticosteroids (ICS). ICS monotherapy should NOT be used in COPD management. ^‡^Surgical therapies may include lung transplantation and lung volume reduction (including with endoscopic valves). ^§^Self-Management Education includes optimizing inhaler device technique and [re-]review, assessment and review of medication adherence, breathing and cough techniques, early recognition of AECOPD, written AECOPD action plan and implementation (when appropriate), promoting physical activity and/or exercise, and other healthy habits including diet and smoking cessation.

**Figure 2:**
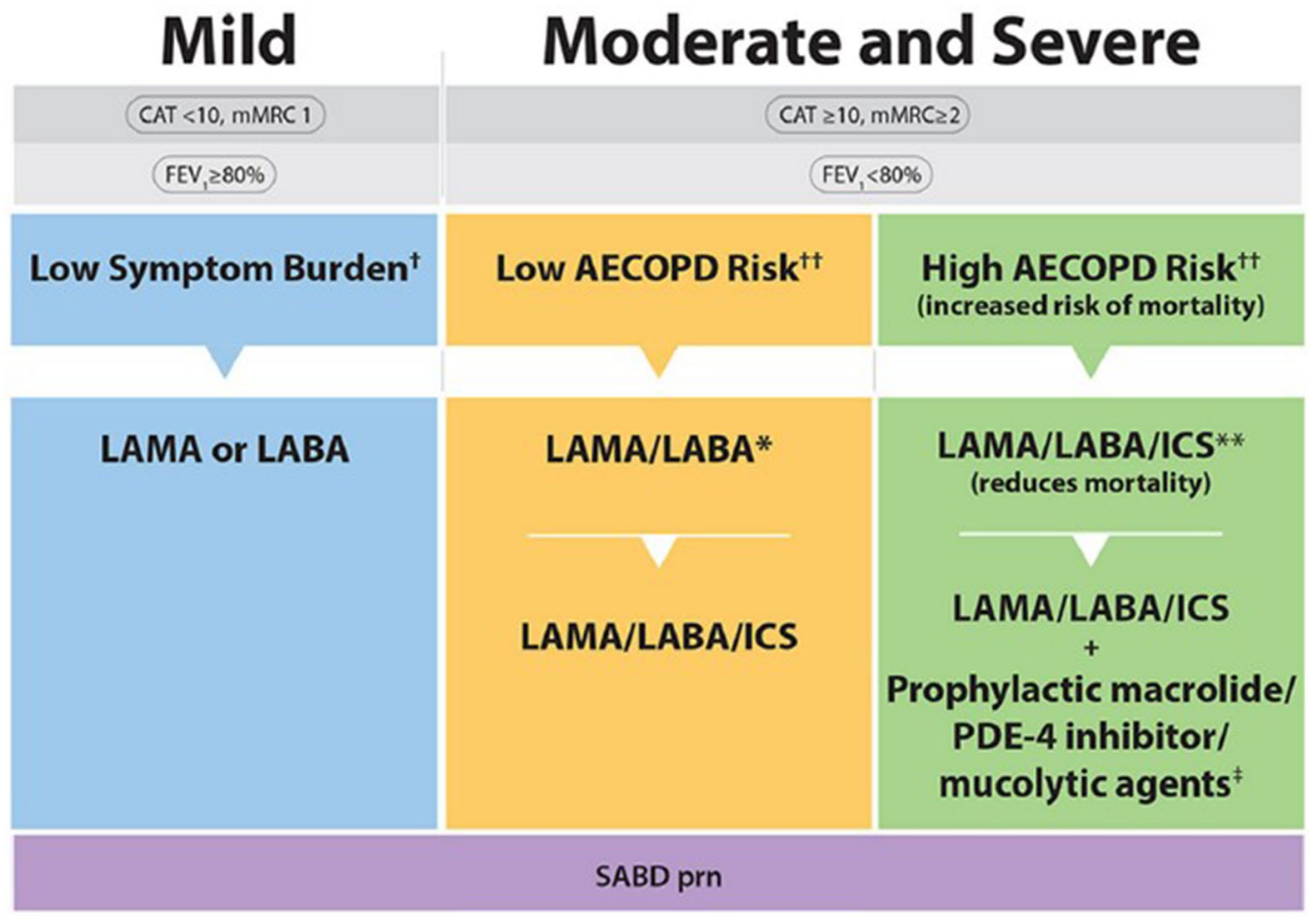
2023 CTS guideline for COPD pharmacotherapy (5). Reprinted by permission of Taylor & Francis Ltd. (https://www.tandfonline.com) on behalf of 2023 Canadian Thoracic Society [from Bourbeau et al. (5)]. CAT, COPD assessment test; mMRC, Modified Medical Research Council; SABD prn, short-acting bronchodilator as needed; AECOPD, acute exacerbation of COPD; ED, emergency department; LAMA, long-acting muscarinic antagonist; LABA, long-acting ẞ2-agonist; ICS, inhaled corticosteroid. ^†^Symptom burden encompasses shortness of breath, activity limitation, and impaired health status. ^††^Individuals are considered at “Low Risk of AECOPD” if ≤1 moderate AECOPD in the last year (moderate AECOPD is an event with prescribed antibiotic and/or oral corticosteroids) *and* did not require hospital admission/ED visit. Individuals are considered at “High Risk of AECOPD” if ≥2 moderate AECOPD *or* ≥ 1 severe exacerbation in the last year (severe AECOPD is an event requiring hospitalization or ED visit). *LAMA/LABA single inhaled dual therapy is preferred over ICS/LABA inhaled combination therapy considering the additional improvements in lung function and the lower rates of adverse events such as pneumonia. ICS/LABA combination therapy should be used in individuals with concomitant asthma. There is no universally accepted definition of concomitant asthma. The 2017 CTS Position Statement on COPD Pharmacotherapy provides guidance on the assessment of patients who may have concomitant asthma. **Triple inhaled ICS/LAMA/LABA combination therapy should preferably be administered in a single inhaler triple therapy (SITT), and not in multiple inhalers, although some patients continue to prefer separate inhalers. ^+^Oral pharmacotherapies in this group include prophylactic macrolide, and phosphodiesterase-4 (PDE-4) inhibitor and mucolytic agents for patients with chronic bronchitis.

The 2023 CTS guideline offers a more streamlined COPD treatment pathway relative to the 2019 guideline (Figure 1) (5, 6). Specifically, the 2023 pharmacotherapy algorithm includes fewer arrows and treatment options, with selection of pharmacotherapy defined by COPD symptom burden, severity of airflow obstruction, and risk of exacerbations (Figure 2) (5). It should be conceived as a menu of treatment options rather than a stepwise approach to pharmacotherapy. Key changes to the 2023 pharmacotherapy pathway are summarized in Table 1.

**Table 1:** Key recommendations for initial pharmacotherapy in patients with stable COPD: comparison of 2023 vs. 2019 CTS recommendations.

	2019 CTS guideline (6)	2023 CTS guideline (5)
Mild COPD, low symptom burden^a^	SABD prn	LAMA or LABA
Moderate and severe COPD Low AECOPD risk^b^	LAMA or LABA	LAMA/LABA^c^
Moderate and severe COPD High AECOPD risk^b^ (increased risk of mortality)	LAMA/LABA or ICS/LABA^e^	ICS/LAMA/LABA^d^ (reduces mortality risk)

Adapted from Bourbeau et al. (5, 6). AECOPD, acute exacerbation of chronic obstructive pulmonary disease; COPD, chronic obstructive pulmonary disease; CTS, Canadian Thoracic Society; ICS, inhaled corticosteroid; LABA, long-acting beta-2 agonist; LAMA, long-acting muscarinic antagonist; SABD, short-acting bronchodilator.

a Symptom burden encompasses shortness of breath, activity limitation, and impaired health status.

b Individuals are considered at “low risk of AECOPD” if ≤1 moderate AECOPD in the last year (defined as an event with prescribed antibiotic and/or oral corticosteroids) and did not require hospital admission/emergency visit; individuals are considered at “high risk of AECOPD” if ≥2 moderate AECOPD or ≥ 1 severe exacerbation in the last year (defined as an event requiring hospitalization or emergency visit).

c LAMA/LABA single inhaled dual therapy is preferred over ICS/LABA inhaled combination therapy considering the additional improvements in lung function and the lower rates of adverse events such as pneumonia. ICS/LABA combination therapy should be used in individuals with concomitant asthma. There is no universally accepted definition of concomitant asthma.

d Triple inhaled ICS/LAMA/LABA combination therapy should be preferably administered in a single inhaler triple therapy (SITT), and not in multiple inhalers, although the CTS acknowledges that some patients continue to prefer separate inhalers.

e Blood eosinophil ≥300 cells/μL in patients with previous AECOPD may be useful to predict a favorable response to ICS combination inhaler.

A substantial change from the previous 2019 CTS guideline is the move toward earlier and more aggressive initial maintenance therapy across all severities of COPD (5, 6). Specifically, the 2023 CTS guideline recommends a long-acting bronchodilator (LAMA or LABA) instead of a short-acting bronchodilator (SABD) in patients with mild COPD and a dual bronchodilator (LAMA/LABA) instead of bronchodilator monotherapy (i.e., LAMA *or* LABA) in patients with moderate or severe COPD at low risk of exacerbations (5). These changes reflect the growing recognition that COPD is a progressive disease supported by a consistent body of evidence demonstrating superior efficacy with more intensive up-front therapy in terms of dyspnea, lung function and reduction in exacerbations, without compromising the overall safety profile (5, 7–9). Moreover, the 2023 CTS guideline specifically favors LAMA/LABA over ICS/LABA as dual bronchodilator therapy based on its greater improvement in lung function and lower risk of side effects (5). An exception is patients with COPD and concomitant asthma, who benefit from the inclusion of an ICS in their treatment regimen (5).

The 2023 CTS guideline now recommends initiation of triple therapy (LAMA/LABA/ICS) in patients with moderate or severe COPD at high risk of exacerbations rather than dual bronchodilator therapy (5) based on strong evidence supporting superior outcomes with triple over dual therapy in this patient population (10, 11). Indeed, the annualized number-needed-to-treat (NNT) for the prevention of 1 moderate or severe exacerbation with triple therapy versus dual bronchodilator therapy is just 3 or 4 (5, 12, 13). The options for initial maintenance therapy in moderate or severe COPD patients at low risk of exacerbation has therefore been simplified to a few fixed dose combination LAMA/LABA inhalers, or a combination of LAMA and LABA single inhaler therapies.

Other changes to the pharmacotherapy pathway include the removal of the 2019 recommendation to use blood eosinophils as a biomarker to predict response to ICS therapy given that there is a lack of prospective, comparative trial data supporting their utility in COPD (i.e., most of the data on blood eosinophils are derived from observational studies and/or *post hoc* analyses) (5). This differs from the Global Initiative for Chronic Obstructive Lung Disease (GOLD) strategy, which suggests a blood eosinophil threshold of >300/μL to identify patients who are more likely to benefit from an ICS in their maintenance treatment regimen based on *post hoc* analyses from the ETHOS and IMPACT studies and some older observational studies; however, GOLD acknowledges that these observations apply at the population level and there is insufficient evidence for blood eosinophils to predict future exacerbations in individual patients (14). Additionally, the recommendation to refer patients with moderate or severe COPD to respirology is no longer included in the 2023 pathway (5). This may be both a recognition of the ability of frontline PCPs to manage the majority of uncomplicated moderate or severe COPD patients and/or the current healthcare system constraints wherein access to respirologists is a barrier in some regions, leading to a vast majority of COPD patients (94%) being managed in the primary care setting (3, 15).

Unchanged from the previous 2019 CTS guideline is the continued focus on the importance of confirming a diagnosis of COPD using post-bronchodilator spirometry and preventing exacerbations, which are a key driver of morbidity, mortality, and healthcare costs in COPD (5, 6, 16). Indeed, it is well established that an AECOPD can trigger a downward spiral of negative consequences including progressive reductions in lung function and physical activity, impaired quality of life and mental health, increased risk of further exacerbations, and ultimately increased mortality (16). Moreover, 30-day readmission rates are high (ranging from 6 to 24% in a meta-analysis of 24 studies) (17) and severe exacerbations are associated with increased risk of all-cause mortality (up to 33% at 12 months and 51% at 5 years) (18). Thus, reduction of exacerbations remains a critical goal from both a personal and societal perspective.

Despite the simplification of the 2023 COPD pharmacotherapy pathway, there remain practical challenges to its implementation in the primary care setting. Notably, a measure of COPD severity is needed to guide appropriate treatment selection (5, 14) and documenting that criteria for medication reimbursement have been met.

A variety of validated questionnaires are available for assessing symptoms and health status in patients with COPD (Table 2). The COPD Assessment Test (CAT) more comprehensively assesses symptoms and how patients are coping with their disease, but the modified Medical Research Council (mMRC) is likely the simplest to implement in primary care and is the most descriptive. Either of these questionnaires could be given to patients to self-complete in the waiting or exam room or sent electronically prior to a scheduled visit.

**Table 2:** Questionnaires for assessment of COPD severity (19).

	Description	Benefits	Limitations
COPD Assessment Test (CAT) (20)	8 items evaluating dyspnea and other elements of health status including impact/disability and coping	Responsive to treatment Evaluates symptoms (dyspnea) and health status Relatively short	More cumbersome to apply in routine practice than the mMRC
Modified Medical Research Council (mMRC) (21)	5 grades evaluating perceived dyspnea	Correlates well with CAT	Only assesses one aspect of the patient experience (dyspnea)
St. George’s Respiratory Questionnaire (SGRQ) (22)	50 items with 76 weighted responses evaluating impaired health in COPD	Comprehensive research scale assessing symptoms, activity, and impact	Lengthy and too complex for routine clinical use
COPD Clinical Questionnaire (CCQ) (23)	10 items evaluating symptoms, functional and mental state	Easy to administer comprehensive health status instrument Responsive to change after smoking cessation	More cumbersome to apply in routine practice than the mMRC

Once dyspnea severity has been evaluated, the risk of exacerbations should be assessed based on historical events. “High risk of exacerbation” is defined as one or more severe exacerbations (i.e., hospitalizations/emergency visits) *or* two or more moderate exacerbations (i.e., prescription of antibiotics or prednisone) in the last year (5). However, exacerbations are frequently under-reported. Indeed, one study suggested that only 32% of patients reported their COPD exacerbations to their PCP (24) and another study found that almost three-quarters of COPD patients had a poor understanding of the term “exacerbation” (25). Given this context, it is not surprising that almost 40% of COPD patients do not take immediate action at the first signs of worsening symptoms (26).

Under-recognition and under-reporting of exacerbations is a considerable barrier. Some practical strategies have been implemented with success to address this problem:

i) Patient self-management tools have been developed to help patients recognize exacerbations and provide guidance on when to seek medical attention for worsening COPD symptoms.
ii) The EXAcerbations of COPD Tool (EXACT) and the COPD Exacerbation Recognition Tool (CERT), are patient-reported outcome measures that were developed and validated for use in clinical trials (27, 28) but they could potentially be applied in the real-world setting.
iii) Digital tools and apps help patients track and record symptoms and exacerbations and can be shared with health providers during routine or acute health visits.

One such tool is MyCOPD,^1^ a digital app designed with input from United Kingdom’s National Health Service (NHS) that has been shown to reduce exacerbations and readmission rates for AECOPD, with savings for the health care system (29, 30). Healthcare providers (HCPs) would need to be aware of such tools in order to understand and appropriately apply the information they provide; there is precedent with this in other chronic disease states notably diabetes, where patients can share information on glucose control collected via devices and self-management apps (31).

There is a need to improve communication channels between HCPs so that when patients seek medical care for worsening COPD, all their care providers are informed about an exacerbation, irrespective of the setting in which patients seek care (e.g., walk-in clinic, pharmacy, acute care clinic, hospital/Emergency department, or primary care clinic). Several digital health solutions could help improve inter-provider communications including dashboards for use of antibiotics or prednisone that can be entered by pharmacists, physicians, or patients themselves; digital monitoring devices and inhalers; and COPD patient registries with resources allocated to be able to track exacerbations, medication use, vaccinations, and more (32, 33). The COMPAS+ Quality Improvement Collaborative in Quebec identified root causes of COPD quality care gaps and proposed a multipronged plan to improve HCP knowledge about COPD and promote interprofessional collaboration and communication (4). One of the solutions they developed was an electronic communication platform for systematic follow-up of COPD patients within 48 h of an exacerbation. This platform automatically alerts all registered members of a patient’s healthcare team of any prescription of antibiotics or prednisone or acute care visit so that timely action can be taken. Less structured and comprehensive interprofessional communication and collaboration strategies that reflect local resources and systems have also been successfully applied. For example, in New Brunswick, a list of 30 keywords that describe AECOPD was compiled, and a RT cross-references the provincial health database daily to flag potential exacerbations in patients seeking care at walk-in clinics. Pharmacists are also well positioned to uncover current exacerbations (e.g., when standing prescriptions for antibiotic or prednisone are filled) or historical exacerbations based on medication reviews. These examples suggest that a variety of communication strategies can help uncover exacerbations and that they need to be tailored to reflect regional resources and practice patterns (4, 34).

A pragmatic challenge to implementing the new COPD pharmacotherapy recommendations is that there can be a lag before reimbursement criteria are aligned with evidence-based guidelines. For example, in some Canadian provinces like Quebec, COPD patients must be initiated on monotherapy (with documentation of a prescription fill) before dual or triple therapy can be prescribed (35). In contrast, Ontario has a limited use (LU) code to facilitate access to dual and triple therapies in COPD (36). The disconnect between reimbursement criteria and provision of guideline-informed care should be a call to action for increased advocacy efforts to improve COPD patients’ access to treatments with demonstrated benefits that could also be cost-saving to the healthcare system.

Finally, the availability of multiple guidelines offering differing recommendations for optimal care of COPD can be a practical challenge in the primary care setting. In this regard, PCPs practicing in Canada are encouraged to follow the CTS guideline, which reflects the realities of the Canadian healthcare context. These are currently among the most up-to-date guidelines on COPD available and they are supported by a rigorous methodology wherein only the highest level of evidence was considered (i.e., randomized controlled trials [RCTs]) with meta-analysis of each clinical question addressed (5). The CTS guideline is internationally recognized and was simultaneously published in the CTS’s own *Canadian Journal of Respiratory, Critical Care, and Sleep Medicine* as well as the American *CHEST* journal (5, 37). The global GOLD strategy is not a guideline *per se* but may be a useful tool that is informed both by published evidence and expert opinion. It is more broadly inclusive of countries with different healthcare systems and resources (14).

*Key take aways*

- *Earlier and more aggressive initial maintenance therapy across all severities of COPD*
- *The NNT to prevent 1 moderate or severe exacerbation with triple therapy compared to dual bronchodilator therapy is between 3 and 4*
- *A measure of COPD severity (*e.g.*, CAT or mMRC score) is necessary to help guide appropriate treatment selection*
- *Risk of exacerbations should be assessed based on historical events*
- *Exacerbations are frequently under-reported*
- *There can be a lag before reimbursement criteria are aligned with guidelines*

*Practical tips for implementation*

- *mMRC grade 2 (walking slower than people of the same age on level ground due to dyspnea) distinguishes mild from moderate or severe COPD*
- *When reimbursement criteria and guidelines are not aligned, consider (i) more frequent follow-ups to evaluate current inhaler response and to allow for timely requests for special authorization for escalation of therapy, and (ii) use samples, depending on availability and regional or institutional regulations*
- *Guidelines are an important tool to help optimize and standardize the delivery of evidence-based care, but they suggest recommendations and should not replace clinical judgment* (38)

### 2.2 Key change #2: mortality reduction

The 2023 CTS guideline is the first to make a formal recommendation on mortality reduction in COPD (5). Specifically, the guideline strongly recommends the use of LAMA/LABA/ICS triple combination therapy over dual bronchodilator therapy with either a LAMA/LABA or LABA/ICS to reduce mortality in patients with moderate or severe COPD who are at high risk of exacerbations (5). This new recommendation is based on data from two large RCTs, IMPACT (39) and ETHOS (40), which reported a consistent benefit using two different single inhaler triple therapy (SITT) regimens. Significant reductions in all-cause mortality and cardiovascular (CV) mortality compared to dual bronchodilator therapy were demonstrated as well as significant improvements in dyspnea, health status, lung function, and exacerbation rates (10, 11).

**Figure 3:**
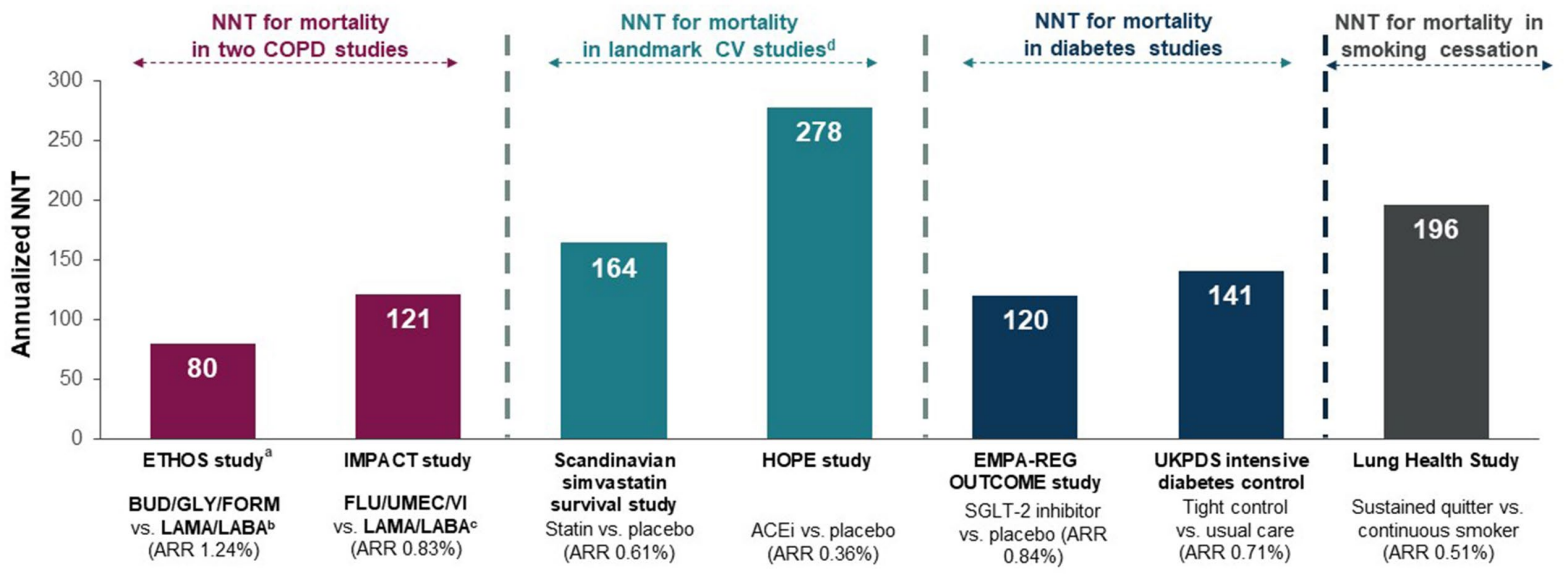
NNTs for the prevention of one annualized death for various medications or strategies for chronic disease management (40, 44, 45). Originally published by, adapted and used with permission from Dove Medical Press Ltd. Numbers-needed-to-treat (NNTs) are calculated from the estimated annualized absolute rate reduction (ARR) and represent the number of patients who need to be treated to prevent one additional death per year. ACEi, angiotensin converting enzyme inhibitor; ARR, annualized absolute rate reduction; BUD, budesonide; COPD, chronic obstructive pulmonary disease; CV, cardiovascular; FLU, fluticasone furoate; FORM, formoterol; GLY, glycopyrronium; LABA, long-acting ẞ2-agonist; LAMA, long-acting muscarinic antagonist; NNT, number-needed-to-treat; SGLT-2, sodium glucose co-transporter 2; UMEC, umeclidinium; VI, vilanterol. ^a^On- and off-treatment deaths with additional data from patients who had incomplete vital status at the time of trial completion; ^b^GLY/FORM; ^c^UMEC/VI; ^d^Landmark CV studies included patients with coronary heart disease and/or diabetes mellitus at high risk of a CV event.

This new recommendation elevates COPD management into the same league as other chronic diseases managed in primary care such as atherosclerotic cardiovascular disease (ASCVD) and diabetes where optimized pharmacotherapy can significantly reduce all-cause mortality. Indeed, the annualized NNT to prevent one death in the COPD studies was 121 in IMPACT (39) and 80 in ETHOS (40), which compares favorably to annualized NNTs for statins (41) and angiotensin converting enzyme inhibition (ACEi) (42) in ASCVD, and intensive glucose lowering vs. usual care (43) and sodium glucose co-transporter 2 inhibitors (SGLT2is) in diabetes (Figure 3) (44). Importantly, the new CTS recommendation empowers PCPs to target mortality as an important goal of COPD management in a well-defined population of high-risk COPD patients based on their exacerbation history (5).

*Key take aways*

- *The 2023 CTS guideline is the first to make a formal recommendation on mortality reduction in COPD*
- *LAMA/LABA/ICS triple combination therapy is strongly recommended over dual bronchodilator therapy with either a LAMA/LABA or LABA/ICS to reduce mortality in patients with moderate or severe COPD who are at high risk of exacerbations*
- *The NNT to prevent 1 death was 80 in ETHOS and 121 in IMPACT*

### 2.3 Key change #3: preference for single over multiple devices

Also for the first time, the 2023 CTS guideline takes a clear position favoring single inhaler combination therapy over a multiple inhaler strategy (5). In the previous 2019 CTS guideline, there was insufficient evidence to favor single vs. multiple device regimens (6). Since then, a number of real-world studies have been published supporting lower exacerbation and mortality rates, increased adherence and persistence rates, and reduced critical technique errors when SITT is initiated compared to multiple inhaler triple therapy (MITT) (46–48). Similar benefits have been reported when switching patients with stable COPD from multiple devices to single device therapy, including a significant reduction in exacerbations (49, 50) and improvements in symptoms, lung function, adherence and patient satisfaction (50). There are convenience benefits to patients when simplifying inhaler regimens to single inhaler devices as well as benefits to HCPs who only need to teach one device.

One potential drawback of single inhaler regimens is the higher unit cost per inhaler device compared to multiple individual-inhaler devices. However, this may be offset by savings with respect to overall medication use and lower prescription fill fees for single vs. multiple devices as well as lower healthcare utilization (51–53). A pragmatic challenge for PCPs is the multitude of devices for inhaled therapies to select from, including the growing number of single inhaler combination therapies, and how to select the one that is best matched to a patient’s skills, preferences, and insurance status and coverage. PCPs need to be familiar with the available devices in order to teach and assess correct inhalation technique.

Inhalation technique is recognized as an important aspect of COPD care (5, 54, 55). Several factors are associated with incorrect inhaler use including multiple devices but also older age, low education level, reduced manual dexterity, lack of inhaler instructions, and cognitive impairment (56). Errors in inhaler handling can lead to Emergency admissions, AECOPD, hospitalizations, and reduced health status (57, 58). Improvements in adherence and inhalation technique with SITT over MITT have been associated with health system cost savings (46, 48). Teaching and assessing inhaler technique can and should be done by all PCPs involved in COPD patient care including FPs, nurses, CREs, RTs, and pharmacists. Repetition is a key principle of adult learning, and regular reinforcement has been shown to significantly improve patients’ inhaler technique, especially among older adults (59, 60). Several forms of inhaler education can be offered to patients to meet their preferences and learning styles, including in person, asynchronous, and videos (available on multiple websites including industry-sponsored sites, local/national/international lung association websites, and the Family Physician Airways Group of Canada^2^); some digital tools such as the MyCOPD app integrate inhaler device tutorials and reminders (29).

*Key take aways*

- *Single inhaler combination therapies are preferred over multiple inhaler strategies*
- *Benefits include convenience for patients and HCPs only need to teach one device*

*Practical tips for regimen simplification*

- *Search electronic medical records (EMR) for patients on multiple inhaler therapies to identify potential candidates for switching to a simplified single inhaler therapy*
- *Proactively switch patients seen in routine follow-up visits after ensuring proper inhaler technique*
- *Describe the multiple benefits of single inhaler regimens to patients who are reluctant to make changes to their treatment plan and/or offer them a trial using a sample, if available*
- *When selecting a maintenance inhaler therapy for stable COPD, consider risk factors for poor inhalation technique including patient beliefs, device or regimen complexity, age, cognition, gender, socioeconomic status and peak inspiratory flow rate (PIFR)* (54, 61)

## 3 Actionable recommendations to address other barriers to enhanced COPD management

The 2023 CTS guideline acknowledges the need for an integrative and comprehensive approach to COPD management that includes confirming a diagnosis of COPD with post-bronchodilator spirometry and implementing both pharmacological and non-pharmacological treatments such as smoking cessation, vaccinations, self-management education, and pulmonary rehabilitation (5). Some of these critical elements of optimal COPD management are beyond the scope of the 2023 CTS guideline update, which is focused on pharmacotherapy. Nonetheless, there continue to exist important barriers to these non-pharmacological aspects of COPD management that have a profound impact on patient care which must be addressed to truly optimize COPD patient outcomes.

### 3.1 Barriers to timely diagnosis of COPD

COPD is a chronic progressive disease, and lung function declines with each successive exacerbation therefore early recognition could prevent or delay lung function impairment (62). However, many adults aged 40 and older with a history of smoking are undiagnosed, and these individuals experience exacerbations and healthcare utilization rates nearing those of individuals with a confirmed diagnosis of COPD (63). There are myriad factors contributing to under-recognition and under-diagnosis of COPD: patient factors such as normalization of symptoms (64), reluctance or nonadherence to undergo recommended testing (65), logistical barriers, and health system and HCP factors including lack of time (66), poor perceived urgency to diagnose COPD (65), limited access to post-bronchodilator spirometry which is necessary for diagnostic confirmation of COPD (5, 14), HCP doubts about clinical utility of spirometry (65), and inadequate compensation for spirometry and its interpretation (65). Key among these barriers to COPD diagnosis are the long wait times and variability in access to spirometry across Canada and other areas of the world (67–70), a problem that was compounded during pandemic shut-downs (71).

The CTS also recommends targeted testing for alpha-1 antitrypsin (A1AT) deficiency in younger individuals and/or in those with worse COPD than would be expected based on their smoking history or a positive family history of A1AT deficiency (Box 1) (72). A1AT deficiency can be assessed with a simple blood test that should be done during periods of clinical stability since levels can fluctuate during illness or stress. Individuals with a positive A1AT test should be referred to respirology for further assessment and treatment.

BOX 1 **CTS recommendations for targeted testing for A1AT deficiency (72)**

Testing is recommended for individuals with COPD who:

- Are diagnosed before age 65 years
- Have a cigarette smoking history of <20 pack-years
- Have a family history of A1AT deficiency

Solving the problem of under-recognition and under-diagnosis of COPD will not be easy and it is likely that a multi-pronged approach that can be tailored to specific regional needs and resources is needed. However, some potential solutions have been proposed and others have been implemented with success. For example, targeted case finding among high-risk individuals could improve early detection and treatment of undiagnosed COPD. A Canadian study called the Undiagnosed COPD and Asthma Population (UCAP) trial examined the feasibility of a case-finding approach to identify adults in the community with undiagnosed respiratory disease (73). Although case finding significantly reduced healthcare utilization and improved patient outcomes, it was resource intensive: trial coordinators called nearly 27,000 symptomatic individuals to identify 595 adults with undiagnosed COPD or asthma. The authors concluded that more efficient case-finding strategies are needed, such as online questionnaires self-completed by symptomatic individuals. Another example involves fee codes to incentivize PCPs to adopt a chronic disease management model for COPD. While this exists in some provinces such as New Brunswick, Manitoba and British Columbia, there are minimum requirements including confirming a diagnosis of COPD with spirometry and seeing the patient a minimum of two times annually by a licensed HCP including at least one visit with the FP claiming the fee code (74). As noted earlier, access to timely spirometry for diagnostic confirmation is a barrier across many regions in Canada. Thus, to make a chronic disease management model feasible for COPD, strategies to improve access to spirometry are needed. There is evidence from the Best Care program in Ontario to support the feasibility and cost-effectiveness of in-office spirometry conducted by a CRE embedded in primary care clinics as part of an integrated disease management (IDM) model of care (75). Some primary care offices have spirometers available; however, remuneration for in-office spirometry is not adequate in all provinces and non-existent in others, and there is a parallel need for educational programs for interpretation of spirometry findings. Expanding the scope of practice of RTs and CREs to allow them to interpret spirometry to establish a diagnosis of COPD could help reduce the burden on FPs and respirologists (76).

Given the high rates of undiagnosed COPD and their associated burden (63, 69), there is a rationale for case finding in individuals with hitherto undeclared symptoms due to denial and/or normalization of symptoms as a consequence of aging or smoking rather than as a disease (77). The Canadian Lung Health Test^3^ is an easy tool for COPD case finding that can be used across primary care and community health settings (Table 3). It focuses on key risk factors for COPD among adults 40 years of age or older who currently smoke or have a smoking history and can identify patients who should be referred for targeted spirometry testing (78). Community pharmacists could potentially play a critical role in identifying patients at high risk of undiagnosed COPD with pharmacy-based case finding programs (79–81).

**Table 3:** The Canadian Lung Health Test: A tool for adults ≥ 40 years of age who are current or former smokers.

	No	Yes
Do you cough regularly?		
Do you cough up phlegm regularly?		
Do even simple chores make you short of breath?		
Do you wheeze when you exert yourself, or at night?		
Do you get frequent colds that last longer than others?		

If you answered “yes” to one or more of these questions ask your doctor about spirometry, a test for COPD.

*Key take away*

- *COPD is under-recognized and under-diagnosed*.

### 3.2 COPD monitoring and follow-up

The CTS guideline advocates for consistent and longitudinal monitoring of COPD symptom burden and exacerbation risk to tailor treatment decisions on an ongoing basis (5). The mMRC is a simple and pragmatic tool that is appropriate for the initial classification of dyspnea severity but it is not responsive to change (21) and thus has limited utility as a monitoring tool to inform decisions to adjust treatment plans. The CAT is a useful tool for identifying trends and changes in symptom control over time, with a minimum clinically important difference (MCID) of 2 points (79).

Despite the clinical utility of such scales for the assessment and monitoring of health status in COPD, there are no clearly defined target thresholds for treatment (i.e., there is no evidence to suggest that patients should be treated to a specific target CAT score of <10 for example) (51).

As noted earlier, digital health interventions and monitoring tools could facilitate patient self-management, with information fed back to HCPs for longitudinal monitoring (32, 33, 80). For example, electronic inhaler monitoring devices can be integrated into or fitted to inhalers to record usage and provide feedback to patients and HCPs. This is a relatively new technology that is being prospectively evaluated in the MAGNIFY COPD pragmatic cluster randomized trial (80). Digital apps can provide patients with access to COPD information, help them monitor signs and symptoms of exacerbations, engage in pulmonary rehabilitation, and even communicate with their HCPs. This field is rapidly evolving, but much could be learned from the field of diabetes where digital health interventions are commonly used by patients and HCPs to track their disease and share information during virtual and in-person visits.

*Key take aways*

- *The CAT is superior to mMRC because it is actionable whereas mMRC is more descriptive*
- *Digital health interventions can help patients and HCPs monitor signs and symptoms of COPD and support self-management*

### 3.3 Access to pulmonary rehabilitation and non-pharmacologic management support

Pulmonary rehab is a guideline-recommended component of the comprehensive management of COPD that includes exercise training, disease education, self-management, and psychosocial support tailored to an individual patient’s needs (5, 6, 14). It has demonstrated benefits on symptoms, mental health status, quality of life and exercise tolerance across all levels of COPD severity (81), as well as reductions in hospitalizations, readmissions, and overall mortality (82). Access to pulmonary rehab is woefully limited in Canada, with some estimates suggesting that <1% of all Canadians with COPD have attended a pulmonary rehab program (83). Given this resource constraint, PCPs caring for patients with COPD are encouraged to refer their patients to other available resources including online programs for tele-pulmonary rehab and education on COPD self-management. *Living Well With COPD* is an online resource for self-management of COPD that was developed in Canada and has been adopted by numerous other countries^4^ (84). There is a growing literature supporting the effectiveness of tele-pulmonary rehab programs, which can be similarly effective as supervised in-person programs (85, 86) and could be an important strategy to address access and resource limitations.

Other important non-pharmacological components of a holistic COPD management plan include identification and minimization of risk factors for exacerbations, vaccinations, smoking cessation, and other healthy lifestyle behaviors (e.g., regular physical activity and healthy eating) (Box 2) (5, 14, 51). In addition to regional and national recommendations on vaccination in patients with chronic disease, the GOLD strategy offers specific guidance on vaccinations in patients with stable COPD (Box 3) (14). Patients should be provided with an individualized action plan, which can be developed in collaboration with CREs and RTs (87).

BOX 2 **Resources to support non-pharmacological management of COPD**

Living Well With COPD https://www.livingwellwithcopd.com/en/home.html

Provincial smoking cessation programs https://www.canada.ca/en/health-canada/services/smoking-tobacco/quit-smoking/provincial-territorial-services.html

Canadian Immunization Guide https://www.canada.ca/en/public-health/services/canadian-immunization-guide.html

COPD action plan template https://cts-sct.ca/wp-content/uploads/2018/03/4915_THOR_COPDActionPlanUpdate_Editable_Eng_v006.pdf

Multiple COPD tools and resources from the Family Physicians Airways Group of Canada website https://www.fpagc.com/tools-resources

BOX 3 **GOLD Strategy recommended vaccinations in patients with stable COPD (14)**

- Influenza
- SARS-CoV-2 (COVID-19)
- One dose of 20-valent pneumococcal conjugate vaccine (PCV20) or one dose of 15-valent pneumococcal vaccine (PCV15) followed by 23-valent pneumococcal polysaccharide vaccine (PPSV23)
- Pertussis (Tdap (dTaP/dTPa) in individuals who were not vaccinated in adolescence
- Zoster
- Respiratory syncytial virus (RSV)

*Key take aways*

- *Pulmonary rehab has demonstrated benefits on symptoms, mental health status, quality of life and exercise tolerance across all levels of COPD severity, and reductions in hospitalizations, readmissions, and overall mortality in those with at least moderate disease*

*Practical tips*

- *When access to pulmonary rehab is limited, three key things PCPs can advise their COPD patients to improve overall health status include i) proper inhaler technique, ii) physical activity, and iii) diaphragmatic breathing* (5, 88)

### 3.4 Identification and management of comorbidities

An estimated 80% of patients with COPD have at least one documented concomitant chronic disease (89). Some of the most common comorbidities are routinely managed in the primary care setting (e.g., CVD, diabetes, obesity, and depression/anxiety) (51) and many share common risk factors (e.g., smoking, aging, and physical inactivity) (14, 90). Other comorbidities include lung cancer, osteoporosis and fracture, cognitive impairment, and gastroesophageal reflux disease, among many others (89, 91). Recognition and management of comorbidities in COPD is critical since they are associated with poorer outcomes including higher morbidity and mortality. Unlike specialists, PCPs manage a patient’s comprehensive wellness and are thus well positioned to assess COPD patients for comorbidities (51).

CVD is increasingly recognized as a critical risk factor for COPD (92, 93) and a leading cause of mortality, particularly in patients with mild and moderate COPD (94, 95). COPD patients should also be screened for lung cancer depending on their risk profile and according to regional standards (96). There is emerging evidence to suggest that a personalized approach to lung cancer screening in COPD patients that takes into account variables including life expectancy and lower thresholds of cigarette smoking in terms of duration and intensity may be beneficial (97).

Finally, in the context of multimorbidity and polypharmacy, simplifying treatment regimens and minimizing the number of medications by using fixed combinations such as dual or triple inhaler therapies can have important benefits for patients in terms of convenience and adherence (51). This approach is aligned with the new 2023 CTS guideline favoring single inhaler dual or triple therapy over multiple inhaler regimens (5).

*Key take aways*

- *Comorbidities are the rule rather than the exception in COPD*
- *PCPs are well positioned to assess and manage some of the most common comorbidities in COPD, notably CVD, diabetes, obesity, and depression/anxiety*

### 3.5 Collaboration between PCPs and specialist care

COPD is a complex disease that is best managed with an integrated disease management (IDM) model (14), which has been shown to enhance outcomes compared to usual care with respect to symptoms, lung function, general health and mental status, and healthcare utilization (98). Several IDM models have been investigated and implemented in COPD, including the Best Care program in Ontario, which has been shown not only to reduce symptoms and exacerbations, but also to reduce health care utilization and generate cost savings (75, 99).

While PCPs can and should manage the majority of patients with COPD, there are situations where specialist care is necessary. Knowing when and who to refer is an important part of triaging patients to maximize the use of specialist services (Box 4).

BOX 4 **Suggested indications for referral of COPD patients to respirology (100)**

- Diagnostic uncertainty
- Age < 40 years and limited smoking history or severe symptoms / disability that is disproportionate to lung function
- Evidence of alpha-1 antitrypsin deficiency (e.g., early COPD onset, unexplained liver disease, family history)
- Signs and symptoms of hypoxemic or hypercarbic respiratory failure
- Severe or recurrent exacerbations and treatment failure
- Severe COPD and disability requiring more intensive interventions (e.g., oral therapy, oxygen, palliative care)
- More intensive comorbidity assessment and management (e.g., sleep disorders, frailty)

## 4 Conclusion

COPD is a complex chronic disease with substantial personal and societal burden. Optimal management strategies continue to evolve, and they reflect the demonstrated benefits of a more comprehensive and integrative approach to chronic disease management, as well as earlier and more aggressive treatment of symptoms and prevention of exacerbations. In 2023, the CTS updated its guideline on the pharmacotherapy of patients with stable COPD (5). This guideline is an important tool to help frontline PCPs to optimize patient outcomes by applying evidence-based principles of care. Key actionable messages from the 2023 CTS guideline for PCPs include (i) the importance of establishing a correct diagnosis as a critical first step for delivery optimal COPD care, (ii) maximizing health status in individuals with COPD by initiating earlier and more aggressive treatment, and (iii) COPD treatments based on disease severity and risk with the goal of preventing exacerbations, which are associated with elevated risk of morbidity and mortality. Improving access to spirometry and pulmonary rehabilitation, adequate vaccination, and developing more effective communication channels between all levels of healthcare providers are important barriers that remain to be addressed for optimized COPD care.

## Author contributions

AK: Conceptualization, Methodology, Writing – original draft, Writing – review & editing. AB: Writing – review & editing. RH: Writing – review & editing. SL: Writing – review & editing. PL: Writing – review & editing. MY: Writing – review & editing.

## Footnotes

^1^ https://mymhealth.com/mycopd

^2^ https://www.fpagc.com/tools-resources

^3^ https://www.lungsask.ca/document/canadian-lung-health-test.pdf

^4^ https://www.livingwellwithcopd.com/en/home.html
